# The role of mortality surveillance in pandemic preparedness and response

**DOI:** 10.2471/BLT.24.292423

**Published:** 2025-01-27

**Authors:** Chalapati Rao, Don de Savigny, Emily Atuheire, Samantha Dolan, Daniel Cobos Munoz, Doris Ma Fat, Joy Ebonwu, Mona Sharan, Anthony Ofosu, Debbie Bradshaw, Rob Dorrington, Erin Nichols

**Affiliations:** a National Centre for Epidemiology and Population Health, Australian National University, 62 Mills Road, Acton ACT 2601, Australia.; b Swiss Tropical and Public Health Institute, Basel, Switzerland.; c Africa Centres for Disease Control and Prevention, Addis Ababa, Ethiopia.; d Bill & Melinda Gates Foundation, Seattle, United States of America (USA).; e Department of Data and Analytics, World Health Organization, Geneva, Switzerland.; f Ghana Health Service, Accra, Ghana.; g South African Medical Research Council, Cape Town, South Africa.; h University of Cape Town, Cape Town, South Africa.; i United States Centers for Disease Control and Prevention, National Center for Health Statistics, United States Public Health Service, Hyattsville, USA.

## Abstract

The coronavirus disease 2019 (COVID-19) pandemic exposed critical limitations in the availability of timely mortality data to inform situational assessments and guide evidence-based public health responses at local, national and global levels. Less than half of the Member States of the World Health Organization (WHO) (73 out of 194) generated the required mortality data. Member States able to meet the sudden demand for real-time data did so through strong public health leadership and strategies for coordinated data acquisition, analysis and dissemination. In most other countries, attempts were made to conduct mortality surveillance but yielded only partial data with limited utility. This experience highlighted the need for a series of strategic shifts to strengthen mortality surveillance programmes in all countries, towards complete recording of deaths and their causes with timely data dissemination. These shifts include modifying systems to enable streamlining of the compilation and use of death records from all sources while meeting the requirements of official registration processes; using electronic protocols for data management and release; and ensuring effective leadership, coordination and data use for public health action. Recently, the Africa Centres for Disease Control and Prevention developed a conceptual framework for strengthening national mortality surveillance and operational guidance for implementation. These activities and resources for improving national mortality surveillance can inform global initiatives to support pandemic preparedness and response programmes. Such initiatives will enable global readiness for early epidemic detection and disease control measure prioritization, while also building routine mortality statistics programmes for population health assessment, health policy and research.

## Résumé

La pandémie de maladie à coronavirus 2019 (COVID-19) a mis en évidence les limites critiques de la disponibilité de données de mortalité en temps utile afin d’informer les évaluations de situation et d’aiguiller les réponses de santé publique fondées sur des données probantes à l’échelle locale, nationale et mondiale. Moins de la moitié des États membres de l’Organisation mondiale de la santé (OMS) (73 sur 194) ont généré les données de mortalité requises. Les États membres capables de répondre à cette demande soudaine de données en temps réel y sont parvenus grâce à une direction forte en matière de santé publique et à des stratégies d’acquisition, d’analyse et de diffusion coordonnées des données. La plupart des autres pays ont tenté de surveiller la mortalité, mais n’ont produit que des données partielles d’une utilité limitée. Cette expérience a mis en évidence la nécessité d’une série de changements stratégiques pour renforcer les programmes de surveillance de la mortalité dans tous les pays, en vue d’un enregistrement complet des décès et de leurs causes et d’une diffusion rapide des données. Il s’agit notamment: de modifier les systèmes pour permettre la rationalisation de la compilation et de l’utilisation des registres de décès provenant de toutes les sources, tout en satisfaisant aux exigences des processus d’enregistrement officiels; d’utiliser des protocoles électroniques pour la gestion et la diffusion des données; et d’assurer une direction, une coordination et une utilisation efficaces des données pour l’action de santé publique. Récemment, les Centres africains de contrôle et de prévention des maladies ont élaboré un cadre conceptuel pour le renforcement de la surveillance nationale de la mortalité et des orientations opérationnelles pour la mise en œuvre. Ces activités et ressources visant à améliorer la surveillance nationale de la mortalité peuvent servir de base à des initiatives mondiales visant à soutenir les programmes de préparation et de réaction aux pandémies. De telles initiatives permettront de se préparer au niveau mondial à la détection précoce des épidémies et à la hiérarchisation des mesures de lutte contre les maladies, tout en mettant en place des programmes de statistiques de routine sur la mortalité pour l’évaluation de la santé de la population, la politique de santé et la recherche.

## Resumen

La pandemia de la enfermedad por coronavirus de 2019 (COVID-19) evidenció limitaciones críticas en la disponibilidad de datos oportunos sobre mortalidad para fundamentar las evaluaciones de la situación y orientar las respuestas de salud pública basadas en pruebas a escala local, nacional y mundial. Menos de la mitad de los Estados Miembros de la Organización Mundial de la Salud (OMS) (73 de 194) generaron los datos de mortalidad requeridos. Los Estados Miembros capaces de satisfacer la repentina demanda de datos en tiempo real lo hicieron gracias a un fuerte liderazgo en salud pública y a estrategias de adquisición, análisis y difusión de datos coordinadas. En la mayoría de los demás países, se intentó realizar una vigilancia de la mortalidad, pero solo se obtuvieron datos parciales de utilidad limitada. Esta experiencia destacó la necesidad de una serie de cambios estratégicos para reforzar los programas de vigilancia de la mortalidad en todos los países, hacia un registro completo de las muertes y sus causas con la oportuna difusión de los datos. Estos cambios incluyen la modificación de los sistemas para permitir la racionalización de la recopilación y el uso de los registros de defunción de todas las fuentes, al tiempo que se cumplen los requisitos de los procesos de registro oficiales; el uso de protocolos electrónicos para la gestión y publicación de datos; y la garantía de un liderazgo, una coordinación y un uso de datos efectivos para la acción de salud pública. Recientemente, los Centros Africanos para el Control y la Prevención de las Enfermedades elaboraron un marco conceptual para reforzar la vigilancia nacional de la mortalidad y unas directrices operativas para su aplicación. Estas actividades y recursos para mejorar la vigilancia nacional de la mortalidad pueden servir de base a las iniciativas mundiales de apoyo a los programas de preparación y respuesta ante pandemias. Estas iniciativas permitirán una preparación global para la detección precoz de epidemias y la priorización de medidas de control de enfermedades, al tiempo que se crean programas de estadísticas rutinarias de mortalidad para la evaluación de la salud de la población, la política sanitaria y la investigación.

## ملخص

كشفت جائحة مرض فيروس كورونا 2019 (كوفيد 19) عن قيود حرجة على توفر بيانات الوفيات في حينها، للتعريف بتقييمات المواقف وتوجيه الاستجابات الصحية العامة القائمة على الأدلة، على المستويات المحلية والوطنية والعالمية. أقل من نصف الدول الأعضاء في منظمة الصحة العالمية (73 من 194) قامت بتجهيز بيانات الوفيات المطلوبة. إن الدول الأعضاء القادرة على تلبية الطلب المفاجئ على البيانات في الوقت الفعلي، تمكنت من القيام بذلك من خلال القيادة القوية للصحة العامة واستراتيجيات الحصول على البيانات، وتحليلها ونشرها بشكل منسق. في معظم الدول الأخرى، تم بذل محاولات لإجراء مراقبة على الوفيات، ولكنها أسفرت عن بيانات جزئية فقط ذات فائدة محدودة. سلطت هذه التجربة الضوء على الحاجة إلى سلسلة من التحولات الاستراتيجية لتعزيز برامج مراقبة الوفيات في جميع الدول، في سبيل التسجيل الكامل للوفيات وأسبابها، مع نشر البيانات في حينها. تشمل هذه التحولات تعديل الأنظمة للتمكن من تبسيط تجميع واستخدام سجلات الوفيات من جميع المصادر، مع تلبية متطلبات عمليات التسجيل الرسمية؛ كذلك التمكن من استخدام البروتوكولات الإلكترونية لإدارة البيانات وإصدارها؛ وضمان القيادة الفعّالة والتنسيق واستخدام البيانات في العمل الصحي العام. وحديثًا، وضعت "مراكز أفريقيا لمكافحة الأمراض والوقاية منها" إطاراً مفاهيمياً لتعزيز المراقبة الوطنية للوفيات، والتوجيه التشغيلي للتنفيذ. إن هذه الأنشطة والموارد لتحسين المراقبة الوطنية للوفيات، يمكنها أن تفيد المبادرات العالمية لدعم برامج الاستعداد للجائحة والاستجابة لها. وستعمل مثل هذه المبادرات على تمكين الاستعداد العالمي للكشف المبكر عن الجائحة، وتحديد أولويات تدابير مكافحة الأمراض، مع بناء برامج إحصاءات الوفيات الروتينية، لتقييم صحة السكان والسياسة الصحية والبحث.

## 摘要

2019 冠状病毒病 (COVID-19) 大流行暴露了在及时提供死亡率数据方面存在严重局限性，这不利于利用此类数据开展情况评估，并指导地方、国家和全球各级的公共卫生机构采取循证应对措施。仅有不到一半的世界卫生组织 (WHO) 会员国（194 个会员国中有 73 个）生成了必要的死亡率数据。能够应突然要求提供实时数据的会员国具有非常强的公共卫生领导力并实施了协调数据采集、分析和传播的战略。其他大多数国家也曾尝试进行死亡率监测，但仅收集了部分数据，实用性很有限。这一经验突出地表明了需要实施一系列战略转变措施，以强化所有国家的死亡率监测方案，实现完整记录死亡事件及其原因，并及时传播数据。这些转变措施包括修改系统，以确保在满足官方登记程序要求的前提下简化所有来源的死亡记录的汇编和使用流程；基于电子协议管理和发布数据；以及确保有效发挥领导作用、开展协调工作和使用数据，以促进实施公共卫生行动。最近，非洲疾病预防控制中心制定了一个概念框架，以加强国家死亡率监测和战略实施相关业务指导。可充分研究这些改善国家死亡率监测的活动和资源，以制定支持大流行防范和应对方案的全球倡议。这些倡议将有利于全球做好准备，确定对早期流行病进行检测和采取疾病控制措施的优先顺序，同时制定可供人口健康评估、卫生政策和研究使用的常规死亡率统计方案。

## Резюме

Пандемия коронавирусной инфекции 2019 года (COVID-19) выявила критические недостатки в наличии своевременных данных о смертности, необходимых для оценки ситуации и принятия научно обоснованных мер в области общественного здравоохранения на местном, национальном и глобальном уровнях. Менее половины государств-членов Всемирной организации здравоохранения (ВОЗ) (73 из 194) представили необходимые данные о смертности. Государства-члены, способные удовлетворить внезапно возникший спрос на данные в режиме реального времени, добились этого благодаря активному руководству в области общественного здравоохранения и стратегиям скоординированного сбора, анализа и распространения данных. В большинстве других стран были предприняты попытки провести наблюдение за смертностью, однако в результате были получены лишь частичные данные, которые не имеют достаточной ценности. Этот опыт выявил необходимость ряда стратегических изменений для укрепления программ наблюдения за смертностью во всех странах, направленных на обеспечение полноценной регистрации смертей и их причин при своевременном распространении данных. В число этих изменений входят: модификация систем, позволяющая оптимизировать процесс сбора и использования записей о смерти из всех источников при соблюдении требований официальных процессов регистрации; использование электронных протоколов для управления данными и их публикации; обеспечение эффективного руководства, координации и использования данных для принятия мер в области общественного здравоохранения. Недавно Африканские центры по контролю и профилактике заболеваний разработали концептуальную схему укрепления национального эпиднадзора за смертностью и оперативное руководство по ее реализации. Эти мероприятия и ресурсы для улучшения национального надзора за смертностью могут стать основой для глобальных инициатив по поддержке программ подготовленности к пандемии и ответных мер. Такие инициативы позволят обеспечить глобальную готовность к раннему выявлению эпидемий и определению приоритетности мер по борьбе с заболеваниями, а также создать программы регулярной статистики смертности для оценки состояния здоровья населения, разработки политики в области здравоохранения и проведения исследований.

## Introduction

Reliable information on deaths by age, sex and cause of death in a population is essential for evidence-based health policy, programme evaluation and epidemiological research. During the earliest phase of infectious disease epidemics, timely mortality data are critical for identifying clusters of deaths from which further investigations can be triggered.^1^ The estimation of excess mortality by continuous tracking of deaths is needed to gauge and monitor disease severity and spread, and also serves as essential evidence for planning and implementation of measures for disease containment, suppression and mitigation.^2^

The coronavirus disease 2019 (COVID-19) pandemic highlighted the lack of availability of such data. During 2020–2021, only 73 of the 194 World Health Organization (WHO) Member States (37.6%) had complete monthly data on deaths by age and sex that could be used to estimate pandemic-related excess mortality (in many cases, only available several weeks after the end of the reference month).^3^ Of the remaining Member States, 37 (19.1%) generated partial data (e.g. either incomplete population coverage or time period) and no mortality data were available for 84 (43.3%).^3^ Mobility restrictions and disruptions in government procedures impeded timely death registration, verification of causes of death and data compilation before release; many public health systems simply did not have the capacity to respond to the sudden increased demand for timely mortality data.^4^ These lack of data highlight the importance of establishing or strengthening mortality surveillance, which is the systematic collection, compilation, analysis and dissemination of the incidence and causes of deaths in defined populations. This continuous cycle of mortality data production in terms of numbers of deaths, mortality rates and patterns in cause of death, along with dissemination of findings to national policy teams, informs public health action and guides real-time responses.^5^^,^^6^ Therefore, mortality surveillance is a vital component of pandemic preparedness programmes.

Mortality surveillance is different from conventional mortality statistics programmes, in that the latter generate annual vital statistics reports of death registration a year or more after the end of the data collection period. Routinely, efficient civil registration and vital statistics systems provide the optimal platform for mortality surveillance, as observed in the countries where timely empirical data were available for monitoring the impact of COVID-19.^7^ In these countries, mortality surveillance activities were initiated or augmented soon after pandemic onset and evolved as the pandemic progressed to meet the demand for more timely information, including the novel cause of death. In many countries, national public health agencies were nominated to source information on deaths notified to subnational or national civil registration authorities. To enhance data completeness and timeliness, registration records were collated with data from other local sources (e.g. health facilities, disease surveillance programmes and funeral services) to compile and release provisional information on all-cause mortality for epidemic surveillance and response.^8^ A range of electronic data solutions were used to capture and transmit data on incident deaths, facilitate record linkage, verify information and support integration to yield consolidated sets of unique death records for specified locations and time periods.^8^

For countries without fully functioning death recording systems, the United Nations (UN) issued guidelines on maintaining civil registration and vital statistics operations during the pandemic.^4^ They also designed and implemented several innovative mechanisms in an attempt to meet the urgent demand for mortality data.^9^^,^^10^ To overcome mobility restrictions, local authorities introduced telephone and online facilities for death reporting, registration and community outreach services, delegating reporting functions to nominated field staff. Similar to countries with sufficient mortality surveillance, local and national public health agencies in low- and middle-income countries implemented processes for electronic compilation of deaths from population registers, health facilities, community health centres, burial sites and mortuaries.^11^^–^^14^ Public health agencies introduced initiatives to strengthen intersectoral coordination of data sharing, death reporting and data management across health and administrative sectors.^15^

Although these interventions led to direct improvements in data availability and enabled more accurate assessments of pandemic impact in Brazil, Colombia, Malaysia, Peru and South Africa,^16^^–^^20^ these ad hoc data compilations were not of sufficient quality in other countries, in terms of timeliness, completeness or adequate disaggregation by sex or age. Therefore these data were of limited value for detailed pandemic mortality analyses.^9^ Furthermore, many of the interventions were not built on institutionalized structures from which the processes could be maintained or sustained after the pandemic. Nevertheless, despite not being entirely successful, the intent and processes for these initiatives at the local level did raise considerable awareness among government agencies, the health systems, civil society and the community about the value of mortality data, and highlighted the importance of strengthening such activities for routine implementation. Hence, it is imperative that such initiatives be sustained and strengthened to support a robust routine mortality surveillance function, based on civil registration and vital statistics processes, as an essential component of public health preparedness and response programmes.

## Strengthening mortality data

### History of initiatives 

The importance of timely mortality data for public health development is well established, and began with the weekly London Bills of Mortality in the seventeenth century for monitoring the occurrence of plague and other epidemics.^21^ These initial experiences led to the establishment of routine mortality statistics programmes embedded within national civil registration and vital statistics systems in many countries in the continents of the Americas and Europe by around 1950, on which mortality surveillance was based.^22^ In the 1960s, the International Program of Laboratories for Population Statistics attempted to strengthen mortality statistics in low- and middle-income countries in Latin America and the continents of Africa and Asia. Initially supported by the United States Agency for International Development,^23^ the programme was discontinued after the withdrawal of external support.^24^ A detailed review of the programme noted implementation challenges that led to the development of a range of analytical demographic techniques to estimate mortality levels and trends using data compiled from censuses and surveys.^24^^–^^26^

The Demographic and Health Surveys programme subsequently started in the 1990s, measuring child and maternal mortality.^27^ In the interim, large countries such as Bangladesh, China and India established sample reporting systems to generate representative statistics, while Honduras, Jamaica, Mexico, Philippines and Thailand undertook long-term national initiatives to strengthen data availability.^28^^,^^29^ Mortality surveillance was also increasingly used as a tool to monitor the impact of infectious disease outbreaks in localized settings, as well as humanitarian emergencies.^30^^,^^31^ However, there remained a difference in perception of the nature of mortality data systems that ensure death registration to generate routine vital statistics, and that of such ad hoc mortality surveillance activities.^32^^,^^33^

Recognizing the need for well-planned and sustainable initiatives to improve mortality data availability, the United Nations Statistics Division published a series of handbooks during 1998–2014 to guide Member States in strengthening civil registration and vital statistics systems.^34^ These handbooks cover legal and regulatory perspectives, management functions, operational aspects of birth and death registration, and the production of vital statistics. These were further updated during 2018−2023 to account for developments in data use and technology, along with the introduction of the United Nations Legal Identity Agenda, which focuses on birth registration and census data collection.^35^ Although there existed a background objective for the division in systems strengthening to enhance data availability, there was no clear link to public health programmes and/or emergency response activities. Consequently, there remained considerable gaps in national mortality data availability as identified by the WHO Global Burden of Disease Study for 2000, which resorted to statistical modelling to fill data gaps for country-level mortality estimation.^36^

WHO subsequently launched the Health Metrics Network in 2005, with the aim of developing a standard approach and framework for strengthening national mortality data systems.^37^ The network developed standardized civil registration assessment tools, and conducted case studies on innovative information technology solutions during 2009–2012 for the compilation, processing and analysis of cause-specific mortality data.^38^ The assessment tools were used by several countries to gain a better understanding of the structural and operational strengths and weaknesses of national mortality data systems.^39^^–^^41^ However, the findings and recommendations of these assessments did not translate into system strengthening interventions, and there was little impact on overall mortality data availability and quality.^42^ There were also initiatives to establish sentinel or sample-based mortality surveillance activities in several countries but, because of funding and other administrative constraints, these were of limited utility with regards to generating reliable population-representative mortality data.^43^^–^^46^

Since 2015, the international initiative Data for Health has been providing funding and technical support to over 40 low- and middle-income countries for the production and use of mortality statistics, primarily through strengthening civil registration systems.^47^ During its initial phase, the initiative focused on systemic issues such as the legal and administrative framework, business process improvement for death notification and data compilation, strengthened cause of death ascertainment and data quality improvement methods.^48^^,^^49^

### Required strategic shifts 

The COVID-19 pandemic highlighted the critical necessity for strategic shifts in coordinated support for mortality surveillance programmes, ensuring that advancements in civil registration and vital statistics systems could result in the production of timely mortality data for public health use. We classify the required changes in approach into seven domains, as listed below (Table 1). 

**Table 1:** Domains in which strategic shifts are needed to develop mortality surveillance systems for pandemic preparedness and response

Domain	Conventional approach	Strategic imperatives for mortality surveillance
Purpose and utility of data	- Population health assessment - Monitoring United Nations’ and national development goals	- Early warning systems - Epidemic monitoring - Evidence for policy and response
Data sources	- Civil registration and vital statistics systems - Disease surveillance programmes - Retrospective household surveys - Mortality modules in population censuses	Civil registration and vital statistics processes applied to report, register and certify deaths identified by all local death recording systems, including: - health sector (facilities, disease surveillance) - civil society (households, funeral services, welfare agencies, insurance) - other administration (identity management, police) - use of population-representative sample-based platforms where appropriate
Timelines for death reporting and data release	- Event notification timelines up to 3 months for civil registration and vital statistics systems - Varying recall periods for surveys - Annual mortality data reports from civil registration systems, often with 12–36-month delay	- Mandated notification of deaths of public health concern within 3–7 days - Use of provisional data for public health purposes - Monthly or quarterly bulletin for routine monitoring of mortality trends - More immediate data release (daily or weekly) during health emergencies
Data modernization and management	- Physical or scanned digital archive - Periodic transmission of data summaries - Centralized statistical analysis and dissemination	- Digitization of death records at point of capture with unique record identifiers - Real-time transmission of complete individual records - Integrated databases (including tools for record verification) - Decentralized analysis and dissemination
System governance	- Civil registration and vital statistics authorities - Information, planning and/or development offices	- Coordinated linkage between data producers (e.g. civil registration and vital statistics authorities, health facilities, burial sites and/or morgues) and national public health agency (technical lead)
Technical support	- National statistics offices - National and international academic institutions - United Nations agencies	- National public health institutes and academic and/or research institutions - District-level universities - Continental and/or regional public health entities (e.g. Africa Centres for Disease Control and Prevention) - International technical resources
Development support	Fragmented financial and/or technical support from different agencies for discrete functions such as: - disease- or programme-specific surveillance - legal review of civil registration and vital statistics systems - cause of death ascertainment - mortality coding - data quality and/or outcome indicator analysis	- Support coordinated and aligned to country-specific strategies -Systems-based support for end-to-end protocols (record capture to dissemination) - Mortality surveillance in population samples within broader national strategies - Projects including measurement of mortality indicators as a stated deliverable - Phased approach starting with demonstrations and pilots then scaled up - Rationalized internal and external resources to support broad development strategies

First, a shift in the purposes of data compilation and utility from routine population health assessment and goal monitoring to the need for real-time mortality data. Mortality surveillance is essential for early warnings and the monitoring of the incidence and progress of epidemics, as well as guiding the public health response. 

Second, instead of compiling health data from retrospective population surveys, disease surveillance programmes and censuses, the demand for more timely mortality data requires the harnessing and integrating of all local data sources, preferably collected in alignment with national civil registration and vital statistics processes.^50^ Where official death notification to the local registration office is delayed (e.g. in emergencies), a minimum set of essential variables for each death could be recorded and complete individual records transmitted directly to the mortality surveillance system, while maintaining routine reporting processes. Non-formal approaches, such as establishing reporting pathways from religious authorities, mortuaries and burial grounds, were successfully used to improve data compilation during the pandemic.^13^ Where routine and timely data collection is not possible at a national scale, population-representative sample-based platforms for reporting deaths and their causes using verbal autopsy methods could be implemented to support data needs.

Third, to improve timeliness, timeframes for reporting deaths and releasing information could be reduced. In most countries without fully functioning civil registration systems, death reporting timelines range from a week to 3 months; these would need to be reduced to 3–7 days for event identification and 1 day for public health authority notification.^1^ The existing public health approach has been to produce annual data reports at least a year after the reference period; however, a surveillance programme could establish regular monthly or quarterly bulletins for monitoring mortality trends and causes of death of public health concern.^34^ During declared public health emergencies, a bulletin compilation and release timeline could be reduced to daily or weekly, supported by regulations permitting provisional data for public health purposes.

Fourth, the modernization of data collection and management is essential to enable efficient and timely data compilation from varied sources and real-time transfer to data users. Timely reporting requires electronic data capture at the source, with data transmission according to national protocols for data sharing and privacy. Although the identity of the deceased is necessary for registration processes, record matching and integration across sources to prevent duplication, all data should be deidentified before statistical analysis. Data integration and flows could be supported through carefully designed system architecture to ensure the relevant data reach the appropriate data users. For better accuracy, record matching across sources could be done at the local level by personnel familiar with the community and contexts. Digital tools for data compilation, analysis and dissemination of decentralized surveillance findings would be useful for displaying cross-sectional results, time trends and other comparative analyses, while providing access across multiple levels of the health system.

Fifth, a shift in system governance from registration authorities and development offices is needed for mortality surveillance. Given the primary role of the health sector in the identification of deaths and their causes, in holding technical responsibilities for data management and analysis, and in ensuring appropriate data interpretation to guide policy and programmatic responses, the national public health leadership could coordinate a surveillance programme.^51^ Administrative support and oversight could be provided by other agencies and coordinating committees responsible for data from other sources, including the civil registration system, health sector surveillance programmes, academia and civil society organizations. Uncoordinated data collection efforts by different ministries could have adverse implications in terms of data duplication or gaps in availability of causes of death. A programme strategy and business continuity plan could help to avoid disruptions to death reporting and data use during public health emergencies. 

Sixth, technical support is mandatory at all levels for surveillance system design and development, capacity-building, monitoring and data interpretation for policy purposes. Previously provided by national statistics offices and UN agencies, such services could be delivered through public health academia as well as local tertiary educational institutions with departments for statistics, epidemiology, demography, basic sciences and humanities. Leveraging recent pandemic experience, public health emergency operations centres could serve as technical nodes for monitoring mortality trends and guiding response.^52^

Finally, there is a strong need for coordinated country-level development support across UN organizations, intergovernmental agencies, multilateral and bilateral development partners, and philanthropic agencies. Most international civil registration development initiatives over the past decade have largely focused on birth registration and legal identity management, with the relative neglect of death registration and mortality statistics programmes. Integrated disease surveillance systems have often had discrete disease-specific data compilation efforts, with no emphasis on reporting all deaths in the population. A common observation is that individual donors support separate small-scale development projects that focus on strengthening particular aspects of mortality data systems or data compilation for a specific purpose, without any harmonization towards a common objective. Various discrete development activities should be synchronized within a coordinated systems approach designed to generate complete, timely and reliable mortality data by sex, age and causes of death for a defined area or population. National mortality surveillance committees could ensure that technical support activities align with national priorities for mortality data in the correct sequence. For example, the use of development resources to provide training on mortality coding should be based on a national strategy for implementation of medical certification of cause of death in sufficient scale, generating the data that need to be coded. 

### Operational strategies 

**Fig. 1:**
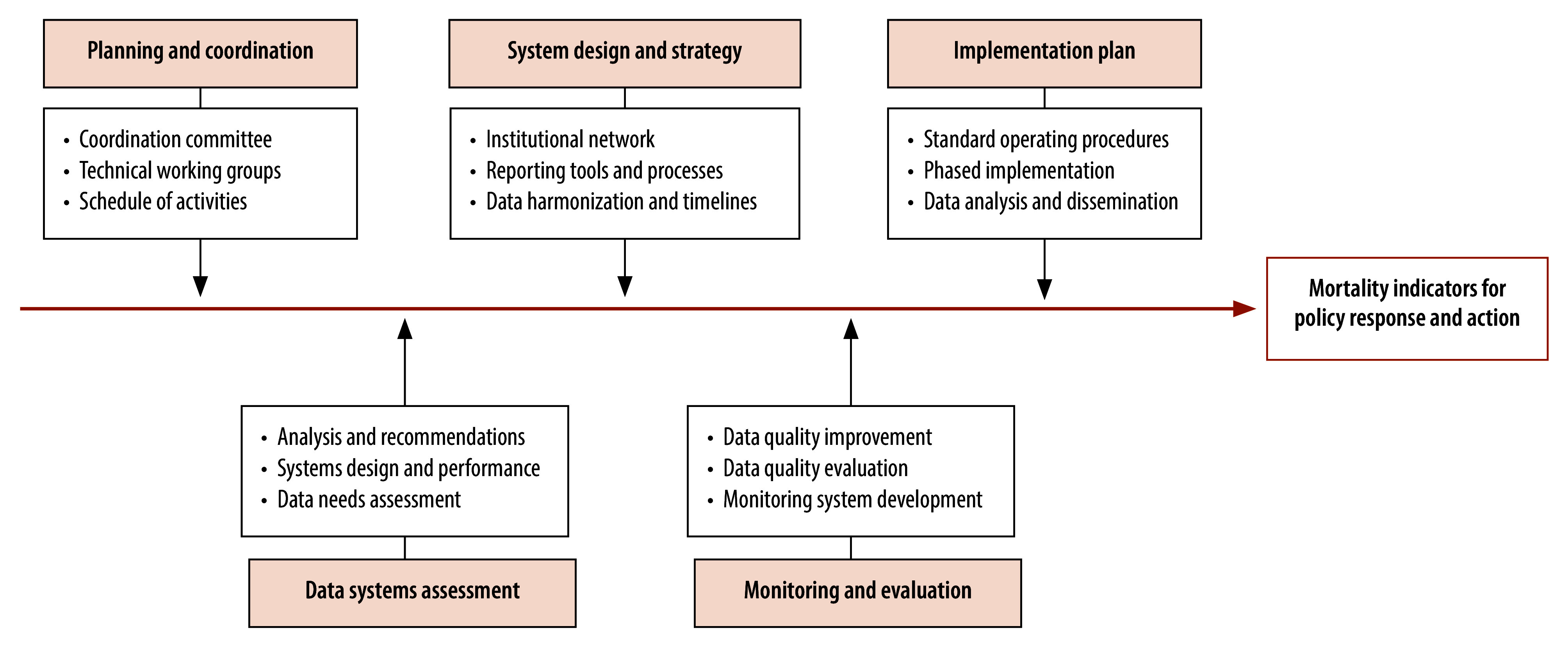
Stages and activities in the development of a national mortality surveillance system to guide public health policy and response

Although gaps in data availability exist in all regions of the world, they are particularly concerning in the WHO African Region in which only four of the 47 Member States were able to report timely data to guide pandemic response.^3^^,^^53^^,^^54^ Death registration systems are dysfunctional in most countries in the African region, where mortality statistics are compiled from fragmented and uncoordinated collection from multiple sources.^55^ To address these data shortcomings, the United Nations Economic Commission for Africa has proposed several initiatives over the past decade.^55^^–^^57^ Building on these initiatives, and driven by the urgent need for timely mortality data, the Africa Centres for Disease Control and Prevention has developed a continental framework for strengthening national mortality surveillance, as well as an operational guide to support its implementation.^5^^,^^58^ The framework and the guide comprehensively address the abovementioned strategic shifts from theoretical and practical perspectives, and provide a detailed road map of mortality surveillance design and implementation activities. Such guidance is relevant and applicable to countries in all regions of the world facing similar challenges. Fig. 1 depicts the broad activities comprising the operational strategy for strengthening timely and reliable mortality data availability globally.

**Fig. 2:**
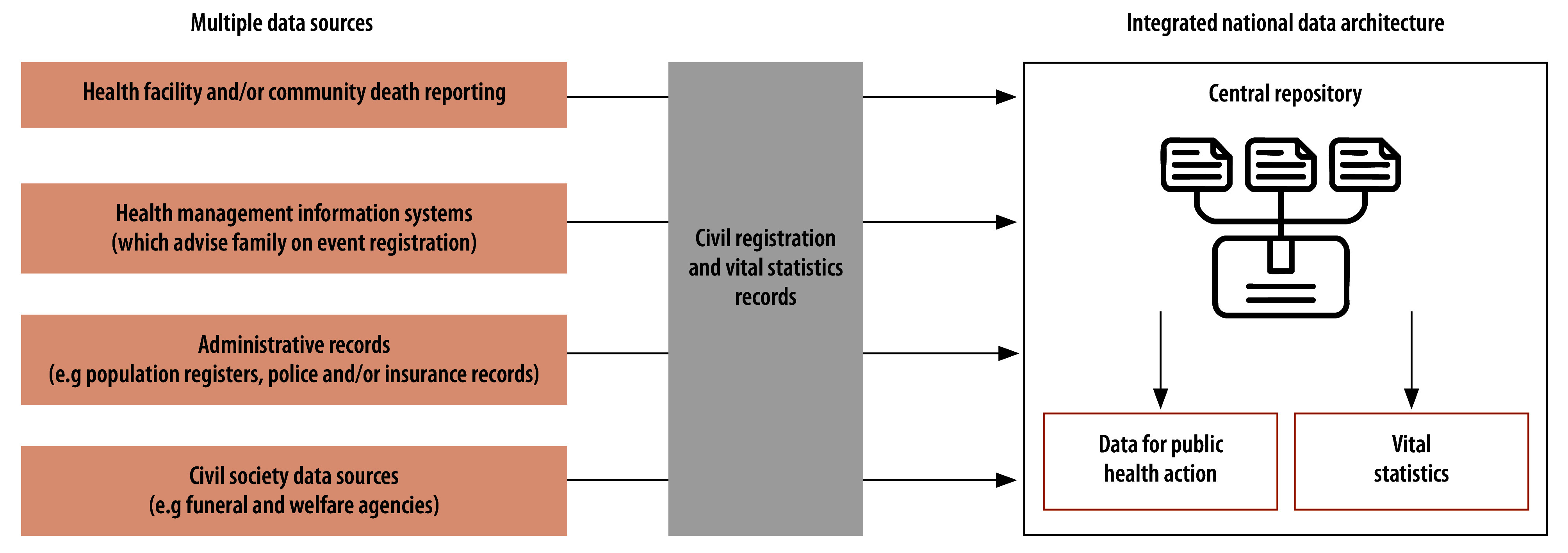
Integrated national mortality data architecture for mortality surveillance, civil registration and routine vital statistics

Assessment of existing mortality data systems is a key primary step, including a review of variables recorded for each death (Box 1). In countries where civil registration and vital statistics systems are not fully functional, the planned mortality surveillance intervention could serve as a basis for establishing standard operating procedures for reporting both community and facility deaths, along with protocols for registration of deaths occurring under suspicious circumstances. The surveillance programme design should integrate death reporting processes across data sources, to ensure that all identified deaths (with unique identifiers where available) from each source are notified to local authorities using official registration protocols. Digitization of individual records and streamlined pathways for data transmission are essential for efficient data management and timely data availability from a central repository (Fig. 2).

Box 1 **Variables to be recorded for each death during infectious disease epidemics**

**Essential variables:**

- Identity, demographic data, including name (for verification purposes), date of birth (or age in completed years), sex, address of usual residence and national identification number (if available);
- event data, including date and address of death occurrence, place of death (home or health facility), name of health facility and date of registration in civil registry; and
- cause(s) of death, including medically certified death, multiple causes (with automated or manual rules-based selection) with duration, cause as determined by verbal autopsy and source of diagnosis, or cause as reported by household.

**Optional variables:**

- other health-related data, including epidemic mortality surveillance variables, such as diagnostic confirmation, vaccination status and access to health care during terminal illness (requiring additional data collection from health information systems where available, or triangulation with multiple data sources).

The importance of monitoring system development according to established timelines cannot be overemphasized. A national coordination committee could establish technical groups to oversee different activities and plan interventions to maintain progress. Surveillance data could be monitored for data timeliness and consistency, with dissemination of primary results through programmed dashboards. In addition, periodic field studies could be conducted to evaluate data reliability and validity, along with the use of more detailed analytical techniques to derive adjusted mortality indicators.^59^^,^^60^ A phased approach to implementation, including the use of representative population samples, could ensure interim data availability (including provisional data) to assess mortality trends and guide policy responses. To this end, governments in Malawi, Mozambique, United Republic of Tanzania and Zambia introduced sample mortality surveillance systems in 2019. The objectives and activities of initiatives across the WHO African Region could be informed by the Africa Centres for Disease Control and Prevention framework and guide.^5^^,^^58^^,^^61^

## The way forward

Although the monitoring of infectious disease mortality is currently a priority, complete and timely data are also required to address the burden from noncommunicable diseases, injuries and a range of health risk factors.^42^ There is a need for a coordinated approach across the various fragmented initiatives supported by different development partners to strengthen the collection of mortality data at multiple levels. It is hoped that one of the key lessons from the pandemic – the importance of reliable data on disease incidence and mortality outcomes for epidemic management – accelerates the improvement of mortality data availability and its use by civil registration systems and public health agencies in all countries.

To achieve this purpose, development activities for mortality statistics programmes need to be reoriented to address the current challenges in improving data availability. Although the international community has developed standardized frameworks and tools for strengthening mortality data systems, there is also a need for country-specific adaptations of the common frameworks into well-supported national action plans that establish systems and structures to address the surveillance elements of data quality, timeliness and use. National action plans can coordinate development assistance and guide implementation projects through a phased approach, such that experiences and lessons from pilot projects in population clusters are used to guide scaled activities across the country. 

The WHO Global Strategic Preparedness, Readiness and Response Plan includes collaborative surveillance as one of its core components; countries must develop stronger data collection and reporting systems for COVID-19 cases, hospitalizations and deaths stratified by age, underlying conditions and vaccination status.^62^ The Global Health Security Agenda of the United States government and the Integrated Disease Surveillance and Response strategy in the WHO African Region also cite the importance of mortality surveillance in identifying clusters of deaths for public health preparedness and response.^63^^,^^64^ Such objectives can only be met through a more broad-based, inclusive and systemic approach to mortality surveillance development, as described here. Meeting such objectives will require a paradigm shift towards collaborative activities with improved coordination between national data producers and data users, international technical agencies and development partners, supported by well-planned investments as part of pandemic preparedness. In conjunction with the rational use of resources, such activities would help to achieve the common objective of timely mortality data availability and use for epidemic detection and management, as well as the long-term goal of strengthened national death registration and mortality statistics programmes.
